# The complete chloroplast genome sequence of *Cinnamomum longipetiolatum*

**DOI:** 10.1080/23802359.2019.1698994

**Published:** 2019-12-12

**Authors:** Yuan Zheng, Yana Luo, Yunqing Li, Yi Wang

**Affiliations:** a Laboratory of Forest Plant Cultivation and Utilization, Yunnan Academy of Forestry, Kunming, People’s Republic of China;; b College of Forestry, Southwest Forestry University, Kunming, People’s Republic of China

**Keywords:** *Cinnamomum longipetiolatum*, chloroplast, Illumina sequencing, phylogenetic analysis

## Abstract

The first complete chloroplast genome (cpDNA) sequence of *Cinnamomum longipetiolatum* was determined from Illumina HiSeq pair-end sequencing data in this study. The cpDNA is 158,603 bp in length, contains a large single copy region (LSC) of 89,386 bp and a small single copy region (SSC) of 18,181 bp, which were separated by a pair of inverted repeats (IR) regions of 25,511 bp. The genome contains 129 genes, including 84 protein-coding genes, eight ribosomal RNA genes, and 37 transfer RNA genes. The overall GC content of the whole genome is 39.0%, and the corresponding values of the LSC, SSC, and IR regions are 37.7%, 33.9%, and 43.0%, respectively. Further phylogenomic analysis showed that *C. longipetiolatum* clustered in a clade in genus *Cinnamomum*.

*Cinnamomum longipetiolatum* is the species of the genus *Cinnamomum* within the family Lauraceae. It is distributed in the south and southeast of Yunnan in China. *Cinnamomum* is used as spice and medicine (Sriramavaratharajan et al. 2016). *Cinnamomum* essential oils possess many biological activities such as anti-microbial, anti-parasitic, anti-inflammatory, antioxidant, antihyperglycemic, anti-diabetic, antitumour, and so on (Ranasinghe et al. 2013). Furthermore, camphor has been reported in the *C. longipetiolatum* (Thang et al. 2008). So, *C. longipetiolatum* is an economically significant plant having great exploitation potentiality; however, there has been no genomic studies on *C. longipetiolatum.*

Herein, we reported and characterized the complete *C. longipetiolatum* plastid genome (MN698965). One *C. longipetiolatum* individual (specimen number: 5309270720) was collected from Cangyuan, Yunnan Province of China (23°18′43″ N, 99°14′52″ E). The specimen is stored at Yunnan Academy of Forestry Herbarium, Kunming, China, and the accession number is YAFH0012762. DNA was extracted from its fresh leaves using DNA Plantzol Reagent (Invitrogen, Carlsbad, CA, USA).

Paired-end reads were sequenced using Illumina HiSeq system (Illumina, San Diego, CA). In total, about 20.2.4 million high-quality clean reads were generated with adaptors trimmed. Aligning, assembly, and annotation were conducted by CLC de novo assembler (CLC Bio, Aarhus, Denmark), BLAST, GeSeq (Tillich et al. 2017), and GENEIOUS v 11.0.5 (Biomatters Ltd, Auckland, New Zealand). To confirm the phylogenetic position of *C. longipetiolatum*, other four species of genus *Cinnamomum* from NCBI were aligned using MAFFT v.7 (Katoh and Standley 2013). The Auto algorithm in the MAFFT alignment software was used to align the five complete genome sequences and the G-INS-i algorithm was used to align the partial complex sequences. The maximum-likelihood (ML) bootstrap analysis was conducted using RAxML (Stamatakis 2006); bootstrap probability values were calculated from 1000 replicates. *Alseodaphne gracilis* (MG407593) and *Alseodaphne huanglianshanensis* (MG407594) were served as the out-group.

The complete *C. longipetiolatum* plastid genome is a circular DNA molecule with the length of 158,603 bp, contains a large single copy region (LSC) of 89,386 bp and a small single copy region (SSC) of 18,181 bp, which were separated by a pair of inverted repeats (IR) regions of 25,511 bp. The overall GC content of the whole genome is 39.0%, and the corresponding values of the LSC, SSC, and IR regions are 37.7%, 33.9%, and 43.0%, respectively. The plastid genome contained 129 genes, including 84 protein-coding genes, 8 ribosomal RNA genes, and 37 transfer RNA genes. Phylogenetic analysis showed that *C. longipetiolatum* clustered in a unique clade in genus *Cinnamomum* (Figure 1). The determination of the complete plastid genome sequences provided new molecular data to illuminate the genus *Cinnamomum* evolution.

**Figure 1.**
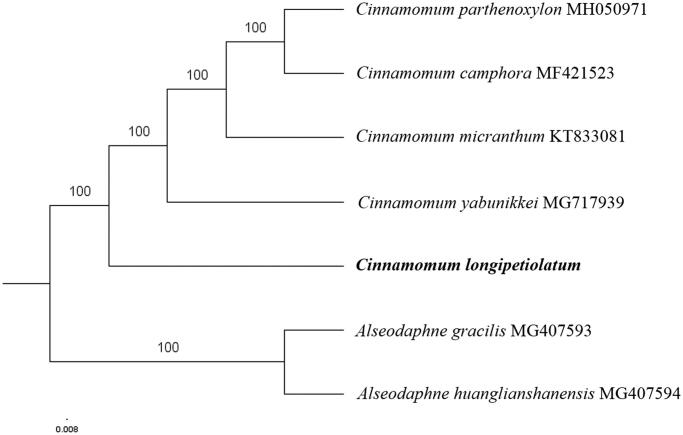
The maximum-likelihood tree based on the 5 chloroplast genomes of *Cinnamomum* genus. The bootstrap value based on 1000 replicates is shown on each node.
